# Association of whole blood essential metals with neurodevelopment among preschool children

**DOI:** 10.1038/s41390-024-03729-9

**Published:** 2024-11-16

**Authors:** Ying Shen, Wanting Zhang, Huyi Jin, Fanjia Guo, Mingjuan Jin, Guangdi Chen

**Affiliations:** 1 Department of Child Health Care, Children’s Hospital, Zhejiang University School of Medicine, National Clinical Research Center for Child Health, Hangzhou, China; 2 Department of Public Health, Zhejiang University School of Medicine, Hangzhou, China; 3 Jiaxing Center for Disease Control and Prevention, Jia Xing, China; 4 Department of Environmental Health, Zhejiang Provincial Center for Disease Control and Prevention Hangzhou, China

## Abstract

**Background:**

Essential metals may play roles in neurodevelopment. The aim was to evaluate the associations of magnesium (Mg), iron (Fe), copper (Cu), and zinc (Zn) levels with neurodevelopment among preschool children.

**Methods:**

The medical records of eligible children enrolled between January 2019 and July 2022 were retrospectively reviewed for required information. The quantitative measurement of metals was conducted using atomic absorption spectroscopy, while screening of neurodevelopment was performed using the Ages and Stages Questionnaire. Modified Poisson regression and Bayesian kernel machine regression (BKMR) analyses were used to evaluate the prevalence ratio (PR) of their independent and joint associations.

**Results:**

662 (14.8%) children were found to have possible neurodevelopmental delays. Modified Poisson regression showed that Mg, Cu, and Zn levels were independently and negatively associated with the risk of neurodevelopmental delay. The PRs (95% CIs) for per log_2_ increment of the above metals were 0.35 (0.19–0.62), 0.57 (0.42–0.77), and 0.63 (0.42–0.96). These negative associations were more pronounced in the gross motor and personal-social domains while considering the concrete five domains. BKMR showed a negative association of metal mixture with the risk of neurodevelopmental delay.

**Conclusion:**

Mg, Cu, and Zn were inversely associated with neurodevelopmental delay. Sufficient essential metal levels are important for neurodevelopment.

**Impact:**

- Essential metals play a key role in neurodevelopment. The association of essential metal mixture with neurodevelopment is relatively scarce.
- Preschool children with possible neurodevelopmental delay are found to have lower Mg, Cu, and Zn levels than their counterparts.
- Single Mg, Cu, Zn levels, and elevated essential metal mixture are negatively associated with the risk of possible neurodevelopmental delay.

## Introduction

Preschool children are at a critical stage of neurodevelopment. Children are deemed delayed when they fall behind their peers in reaching neurodevelopmental milestones.^1^ Globally, approximately 52.9 million children under the age of five suffered from developmental disorders (including intellectual and behavioral functions),^2^ and 10–15% of children had neurodevelopmental problems.^3^ Neurodevelopmental delay can hinder the healthy growth of individuals, resulting in an elevated susceptibility to autism spectrum disorders (ASD) or attention deficit hyperactivity disorder (ADHD), a decline in future quality of life, and a reduction in personal socioeconomic achievement.^4,5^ The Ages and Stages Questionnaire, Third Edition (ASQ-3, Chinese version), is widely used as an effective questionnaire for screening the neurodevelopmental status among children aged 1 to 66 months.^6,7^

Both genetic and environmental factors play a key role in neurodevelopment. Essential metals widely exist in nature and have a critical impact on human health through eating food, drinking water and other means.^8,9^ Among these, magnesium (Mg), iron (Fe), copper (Cu), and zinc (Zn) are indispensable for neurodevelopment in preschool children.^10–13^ These metals are important cofactors in the metabolism of various enzymes, participate in the synthesis and transport of neurotransmitters, are involved in the regulation of oxidative stress and inflammation, and promote the normal physiological functions of the nervous system.^14–16^ Therefore, one of the pragmatic approaches to enhancing neurodevelopment is through the provision of moderate essential metals.

Currently, some previous studies have described the distribution of essential metals and investigated the association of a single metal with neurodevelopment, but the results have been ambiguous.^17–22^ For instance, a case-control study found that children with ASD had significantly lower urinary Zn levels than healthy controls, which are associated with a reduced risk of ASD,^22^ but a meta-analysis of observational studies on Zn and ADHD found no significant difference in blood and hair Zn levels between healthy and ADHD children.^17^ Nonetheless, multiple exposure to essential metals is a more accurate reflection of real-life situations than single metals, and interactions between these metals may either weaken or strengthen the health effects of individual metals. As a result, the association of multiple exposures to essential metals with neurodevelopment has gradually attracted much attention,^23^ while the evidence is relatively scarce.

The aim of this study was to evaluate the associations of essential metals with neurodevelopment among preschool children at both single and mixture levels and to provide etiological clues and prevention strategies for neurodevelopmental delay.

## Methods

### Study design and subjects

This study was approved by the Institutional Review Board of the Children’s Hospital, Zhejiang University School of Medicine (2021-IRB-185).

Our study retrospectively reviewed the electronic medical records database of subjects who underwent health checkups at the Children’s Hospital of Zhejiang University School of Medicine from January 2019 to July 2022. Subjects were considered eligible in the study if they were (1) 1 to 66 months of age; (2) had data on essential metal levels and neurodevelopmental status from the ASQ-3; and (3) had no neurological disorders or physical disabilities. The exclusion criteria were as follows: (1) duplicate records (records with more information or the earliest ones were retained); (2) no key covariates (age, sex, height or body length, weight). A total of 6 418 records met the inclusion criteria, and after excluding duplicates (*n* = 1 928) and records without key covariates (*n* = 3), 4 487 preschool children were finally included in this study.

### Metal detection

Whole blood samples (4 ml) were obtained by using heparin vacuum blood collection tubes (20 international units of lithium heparin/ml). Before measurements, 40 μl sample was accurately aspirated into 1.96 ml of blood diluent, and thoroughly mixed by vibration for 1 minute. The quantitative measurement of Mg, Fe, Cu, and Zn contents was performed by using flame atomic absorption spectroscopy on a multichannel atomic absorption spectrophotometer-MB5 (Beijing Purkinje General Instrument Company Limited, Beijing, China).^24^ Standard curves were drawn to calculate the concentrations of metals. The detection limits of the instrument for Mg, Fe, Cu, and Zn were all 0.01 μmol/L. The reference intervals of essential metals were consistent with the current standard used clinically, which are shown in Table S1.

### Outcome ascertainment

The ASQ-3 was used for preliminary screening among children aged 1 to 66 months in our study.^25^ The ASQ-3 has different age-based versions, which consist of age-specific neurodevelopmental milestones. Each ASQ-3 contains 30 items across 5 domains: problem-solving, communication, gross motor, fine motor, and personal-social. Additionally, the ASQ-3 also includes 6–10 comprehensive questions to provide more detail about neurodevelopmental concerns without scoring. Parents and their children work together to complete the ASQ-3 for 10–15 minutes. For each item, the parent indicates “Yes” (Yes =10 points) if the child performs the item, “Sometimes” (Sometimes = 5 points) if the child performs occasionally or emerging, and “Not yet” (Not yet = 0 points) if the child has not yet performed. Total and each domain scores were added up by the pediatrician and the scores were compared to age-based cutoffs yielding the categorical risk: on schedule (higher than age-based cutoff), monitored (at age-based cutoff), or possible delayed (lower than age-based cutoff), respectively. In our study, preschool children were considered likely to be neurodevelopmental delay if they were at the categories of monitored or possibly delayed.

### Covariate assessment

Age in months was calculated from the date of birth to the date of checkup. Height was measured using a baby height-measuring board for children under 2 years old and a height-measuring instrument with an accuracy of 0.1 cm for children over 2 years old. Weight was measured using a lever scale (electronic scale for babies) with an accuracy of 50 g. The z-score= (X-Mean)/SD, in which X is the height/weight value, Mean and SD are the mean and standard deviation values of the distribution corresponding to the reference population. The BMI (kg/m^2^) was calculated as weight (kg) divided by height (m) squared and was determined using the age- and sex-specific standardized growth chart according to the World Health Organization (WHO).^26^ Based on the WHO Child Growth Standards, the underweight, overweight, and obese were defined as ≤ the 5th, ≥ 85th and ≥ 95th BMI percentiles, respectively.^27^

### Statistical analysis

To describe the basic characteristics, the categorical variables were presented as median with interquartile range (IQR) defined as the range between the 25th to 75th percentile, and continuous variables were expressed as numbers with percentages. Demographic characteristics were compared between groups using the chi-squared test for categorical variables and the Wilcoxon signed-rank test for non-normally continuous variables. The prevalence ratio (PR), which is the exposure level-specific prevalence proportion divided by the prevalence proportion in the reference category, was used to evaluate the association of metal exposure and the risk of neurodevelopmental delay.

In each essential metal exposure, for the continuous focus, the concentration of each metal was log_2_ transformed to obtain normal distributions. Restricted cubic spline (RCS) was used to quantify the dose-response relationship between a single metal and the risk of neurodevelopmental delay, with the reference set at the 10th percentile and knots placed at the 10th, 50th, and 90th percentile. For the categorical focus, as more than 99% of subjects exhibited Mg, Fe, and Cu levels within the age-specific reference interval (Table S1), they were divided into three categories by tertiles: the lowest tertile (T_1_) as the reference, the medium tertile (T_2_), and the highest tertile (T_3_). Nevertheless, as 31.8% of subjects exhibited Zn level below the age-specific reference interval (Table S1), they were divided into three categories: low-level, ≤ median, and > median for those within the reference interval. And ≤ median category was designated as the comparison. A modified Poisson regression model was used to estimate the association between each metal category and the risk of neurodevelopmental delay, adjusted for age (<1 year, <2 years, <3 years, <4 years, and 4-5.5 years), sex (boy and girl), and BMI (underweight, normal, overweight, and obesity).^28^ Stratified analysis by age, sex, and BMI was performed. The potential impact of unmeasured confounding factors on outcome indicators was evaluated by the E-value.^29^

In essential metal mixture exposure, bayesian kernel machine regression (BKMR) was implemented to detect the overall association of metal mixture with the risk of neurodevelopmental delay and estimate the univariate summaries of changes in the risk for neurodevelopmental delay associated with changes in a specific metal from its 10th to 90th percentile, while all other metals were fixed at a particular threshold.^30^ The BKMR model is given below:

$${Y}_{i}=h({{{\rm{Mg}}}},{{{\rm{Fe}}}},{{{\rm{Cu}}}},{{{\rm{Zn}}}})+{\beta x}_{i}+{e}_{i}$$

The $h()$ function is a exposure-response machine function, $\beta$ represents the effect estimates of the covariates, ${x}_{i}$ represents confounding factors to be adjusted (age, sex and BMI), and ${e}_{i}$ represents residuals. Posterior inclusion probability (PIP) values were calculated, using a threshold of 0.5 to determine the relative importance of each metal in the overall mixture effect.^31^ The model was fitted by the Markov chain Monte Carlo with 10,000 iterations.^32^ The R package “bkmr” was used for BKMR analysis.

All analyses were performed in R software 4.2.2. A two-sided *p* value < 0.05 was considered statistically significant. It has been recognized that there are multiple statistical tests for data arising from individual preschool children. However, as the final multivariable models are to be considered as the main definitive results, all other tests are to be considered only for explorative purposes and not subject to the concerns of multiple testing.

## Results

### Subject characteristics

Table 1 shows the basic characteristics of the 4 487 preschool children. Among them, 3 825 (85.2%) were classified as neurotypical children, while 662 (14.8%) were identified as possible neurodevelopmental delay children. There were statistically significant differences in age (*p* < 0.001), sex (*p* < 0.001), and BMI (*p* = 0.029). The concentrations (median [P_25_, P_75_]) of Mg, Fe, Cu and Zn in the neurotypical group were 1.61 × 10^3^ (1.50 × 10^3^, 1.71 × 10^3^) µmol/L, 8.59 × 10^3^ (8.05 × 10^3^, 9.15 × 10^3^) µmol/L, 20.49 (18.08, 23.29) µmol/L, and 72.00 (63.25, 81.09) µmol/L, and those in the delay group were 1.58 × 10^3^ (1.48 × 10^3^, 1.70 × 10^3^) µmol/L, 8.56 × 10^3^ (7.95 × 10^3^, 9.17 × 10^3^) µmol/L, 19.95 (17.55, 22.83) µmol/L, and 70.97 (61.80, 78.58) µmol/L, respectively. There were significantly lower levels of Mg (*p* < 0.001), Cu (*p* = 0.002), and Zn (*p* = 0.009) in the delay group versus in the neurotypical group, while there was no statistical difference in Fe (*p* = 0.143).

**Table 1:** Basic characteristics in preschool children.

Characteristics	Overall (*n* = 4 487)	Typicality (*n* = 3 825)	Delay (*n* = 662)	*p* value
Age, *n* (%)				
<1 year	555 (12.4)	457 (11.9)	98 (14.8)	<0.001
<2 years	1 245 (27.7)	1 064 (27.8)	181 (27.3)	
<3 years	832 (18.5)	682 (17.8)	150 (22.7)	
<4 years	775 (17.3)	694 (18.1)	81 (12.2)	
4–5.5 years	1 080 (24.1)	928 (24.3)	152 (23.0)	
Sex, *n* (%)				
Boy	2 585 (57.6)	2 134 (55.8)	451 (68.1)	<0.001
Girl	1 902 (42.4)	1 691 (44.2)	211 (31.9)	
Hength/height-for-age z-score, *n* (%)				
<-2	68 (1.5)	54 (1.4)	14 (2.1)	0.392
-2 to 2	4 271 (95.2)	3 645 (95.3)	626 (94.6)	
>2	148 (3.3)	126 (3.3)	22 (3.3)	
Weight-for-age z-score, *n* (%)				
<-2	59 (1.3)	45 (1.2)	14 (2.1)	0.122
-2 to 2	4 295 (95.7)	3 664 (95.8)	631 (95.3)	
>2	133 (3.0)	116 (3.0)	17 (2.6)	
Body mass index (kg/m^2^), *n* (%)				
Underweight	216 (4.8)	177 (4.6)	39 (5.9)	0.029
Normal	3 657 (81.5)	3 142 (82.1)	515 (77.8)	
Overweight	393 (8.8)	318 (8.3)	75 (11.3)	
Obesity	221 (4.9)	188 (4.9)	33 (5.0)	
Metal level (µmol/L), Median (P_25_, P_75_)^*^				
Mg	1.60 × 10^3^ (1.50 × 10^3^, 1.71 × 10^3^)	1.61 × 10^3^ (1.50 × 10^3^, 1.71 × 10^3^)	1.58 × 10^3^ (1.48 × 10^3^, 1.70 × 10^3^)	<0.001
Fe	8.59 × 10^3^ (8.03 × 10^3^, 9.16 × 10^3^)	8.59 × 10^3^ (8.05 × 10^3^, 9.15 × 10^3^)	8.56 × 10^3^ (7.95 × 10^3^, 9.17 × 1 0^3^)	0.143
Cu	20.40 (18.04, 23.24)	20.49 (18.08, 23.29)	19.95 (17.55, 22.83)	0.002
Zn	71.80 (63.00, 80.72)	72.00 (63.25, 81.09)	70.97 (61.80, 78.58)	0.009

^*^(P25, P75) is the inter-quartile range (IQR).

### Association between single essential metal and neurodevelopment

Significantly inverse linear dose-response relationships of log_2_ transformed Mg (*p*_*-overall*_ < 0.001, *p*_*-non-linear*_ = 0.303), Cu (*p*_*-overall*_ = 0.002, *p*_*-non-linear*_ = 0.636), and Zn (*p*_*-overall*_ = 0.048, *p*_*-non-linear*_ = 0.334) values with the risk of neurodevelopmental delay were found by RCS (Figure S1). Table 2 shows that the PRs (95% CIs) for per log_2_ increment estimated by modified Poisson regression were 0.35 (0.19–0.62) for Mg, 0.57 (0.42–0.77) for Cu, and 0.63 (0.42–0.96) for Zn, further supporting the monotonic and linear dose-response relationship. When dividing these metals into categorical variables, the PRs (95% CIs) of T_2_ and T_3_ were 0.79 (0.65–0.96) and 0.74 (0.61–0.91) for Mg, 0.76 (0.62–0.92) and 0.71 (0.58–0.87) for Cu compared to the T_1_ category after adjusting for age, sex, and BMI, respectively. Compared to the ≤ median category of Zn, those in the > median category had a 22% (PR 0.78, 95% CI 0.61–0.99) reduction in the risk of neurodevelopmental delay. Stratified analysis across age, sex, and BMI showed that the associations also kept in accordance with the above results basically (Table S2–S4). Sensitivity analysis was performed to assess the robustness of the above results. The possible effects of unadjusted confounding factors were estimated, and the E-values of Mg, Fe, Cu and Zn were 5.16 (2.61), 2.12 (1.00), 2.90 (1.92), and 2.55 (1.25), respectively, showing that the results were stable.

**Table 2:** Association of essential metal with the risk of neurodevelopmental delay.

Metal	Overall	Problem solving	Communication	Gross motor	Fine motor	Personal-social
Delay/Typicality, *n*	662/3 825	121/3 825	171/3 825	242/3 825	264/3 825	241/3 825
PR (95% CI)^*^						
Mg						
Per log_2_ increment	0.35 (0.19–0.62)	0.48 (0.14–1.59)	0.42 (0.13–1.39)	0.19 (0.08–0.44)	0.38 (0.15–0.98)	0.26 (0.11–0.62)
T_1_	1.00	1.00	1.00	1.00	1.00	1.00
T_2_	0.79 (0.65–0.96)	0.70 (0.44–1.10)	0.63 (0.43–0.94)	0.90 (0.66–1.22)	0.82 (0.60–1.11)	0.78 (0.56–1.07)
T_3_	0.74 (0.61–0.91)	0.69 (0.44–1.08)	0.84 (0.58–1.21)	0.60 (0.43–0.85)	0.81 (0.59–1.11)	0.70 (0.50–0.97)
*p*_*-trend*_	0.005	0.102	0.310	0.003	0.179	0.029
Fe						
Per log_2_ increment	0.72 (0.38–1.35)	1.16 (0.32–4.27)	2.86 (0.88–9.30)	0.20 (0.08–0.51)	0.63 (0.24–1.62)	0.71 (0.26–1.94)
T_1_	1.00	1.00	1.00	1.00	1.00	1.00
T_2_	0.84 (0.68–-1.03)	0.75 (0.47–1.19)	1.24 (0.84–1.81)	0.72 (0.52–0.98)	0.98 (0.72–1.33)	0.87 (0.64–1.20)
T_3_	0.87 (0.71–1.07)	0.97 (0.63–1.48)	1.39 (0.93–2.09)	0.59 (0.43–0.82)	0.91 (0.66–1.25)	0.78 (0.56–1.08)
*p*_*-trend*_	0.205	0.850	0.110	0.002	0.573	0.130
Cu						
Per log_2_ increment	0.57 (0.42–0.77)	0.63 (0.31–1.27)	0.87 (0.50–1.52)	0.52 (0.33–0.82)	0.62 (0.38–1.01)	0.71 (0.45–1.13)
T_1_	1.00	1.00	1.00	1.00	1.00	1.00
T_2_	0.76 (0.62–0.92)	0.89 (0.57–1.41)	0.87 (0.59–1.28)	0.78 (0.57–1.06)	0.79 (0.58–1.08)	0.98 (0.71–1.35)
T_3_	0.71 (0.58–0.87)	0.83 (0.52–1.32)	0.88 (0.60–1.27)	0.67 (0.49–0.93)	0.76 (0.56–1.03)	0.92 (0.66–1.27)
*p*_*-trend*_	0.001	0.432	0.503	0.018	0.085	0.600
Zn						
Per log_2_ increment	0.63 (0.42–0.96)	1.52 (0.70–3.30)	2.27 (1.05–4.89)	0.36 (0.19–0.66)	0.75 (0.37–1.55)	0.47 (0.24–0.91)
Low-level	1.03 (0.84–1.25)	0.76 (0.49–1.18)	0.62 (0.43–0.91)	1.33 (0.97–1.83)	1.08 (0.79–1.47)	1.07 (0.78–1.47)
≤ Median	1.00	1.00	1.00	1.00	1.00	1.00
> Median	0.78 (0.61–0.99)	0.96 (0.57–1.61)	0.77 (0.47–1.26)	0.74 (0.51–1.08)	0.90 (0.61–1.34)	0.79 (0.53–1.17)
*p*_*-trend*_	0.120	0.693	0.394	0.190	0.889	0.148

^*^The prevalence ratios were estimated by modified Poisson regression, adjusted for age, sex, and BMI.

The level of Zn is divided into three groups: Low-level, ≤ Median (Reference), > Median.

*PR* prevalence ratio, *CI* confidence interval, *T*_*1*_ 1st tertile; *T*_*2*_ 2nd tertile, *T*_*3*_ 3rd tertile.

Furthermore, Table 2 also shows the results of five neurodevelopmental domains. There were inverse associations of Mg level with the risk of neurodevelopmental delay in the gross motor (PR for per log_2_ increment 0.19, 95% CI 0.08–0.44) and personal-social (PR for per log_2_ increment 0.26, 95% CI 0.11–0.62) domains. The level of Cu was negatively associated with neurodevelopmental delay only in the gross motor (PR for per log_2_ increment 0.52, 95% CI 0.33–0.82). The level of Zn had potential negative associations with neurodevelopmental delay in the gross motor (PR for per log_2_ increment 0.36, 95% CI 0.19–0.66) and personal-social (PR for per log_2_ increment 0.47, 95% CI 0.24–0.91) domains.

### Association between essential metal mixture and neurodevelopment

**Fig. 1:**
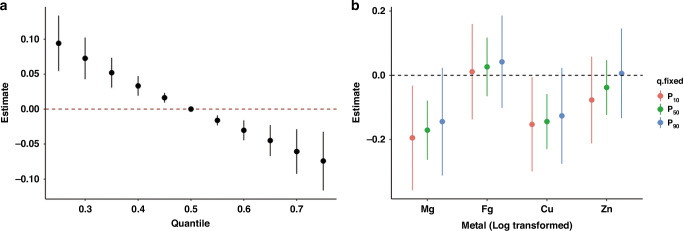
Overall and individual effects of Mg, Fe, Cu, and Zn on the risk for neurodevelopmental delay, by bayesian kernel machine regression (BKMR). **a** Overall effect. BKMR was used to assess the association between essential metal mixture (Mg, Fe, Cu, and Zn) exposure and the risk of neurodevelopmental delay where the four metals are at a specific quantile level compared to the risk when the mixture is at the 50th percentile. **b** Individual effect. BKMR was used to assess the association between individual essential metal exposure and the risk of neurodevelopmental delay when the metal is at the specific adjusted for age, sex and BMI to the risk when all other metals are fixed at their 10th, 50th, or 90th percentile. Models were adjusted for age, sex and BMI.

In the BKMR models, Fig. 1a shows the overall effect of essential metal mixture on the risk of neurodevelopmental delay by comparing the concentrations of the mixtures to those at the 50th percentile for all metals. Compared to the 50th percentile, the risk of neurodevelopmental delay decreased as the concentration of all metals rose when the mixture concentration was at the 55th percentile or higher, whereas the risk increased when the mixture concentration was at the 45th percentile or lower. The PIP values for Mg, Fe, Cu, and Zn were 0.939, 0.293, 0.718, and 0.264, respectively, indicating that the more important contributors to the joint effect of the essential metal mixture were Mg and Cu (PIP > 0.5). Further, Fig. 1b shows the risk of neurodevelopmental delay associated with changes in a specific metal, while all other metals were fixed at a particular threshold. An inverse association was found of Mg and Cu with the risk of neurodevelopmental delay when the other three metals were fixed at their 10th, 50th, or 90th percentile. The association was a bit stronger in Mg than that in Cu.

## Discussion

This study explored the association between essential metals and neurodevelopment among preschool children. Compared to neurotypical children, those with neurodevelopmental delays have lower levels of Mg, Cu, and Zn. Single exposure analysis demonstrated that there were negative associations between Mg, Cu, and Zn levels and the risk of neurodevelopmental delay, particularly in the gross motor and personal-social domains. Multiple exposure analysis indicated that an increased essential metal mixture was associated with a reduced risk of neurodevelopmental delay.

Mg is an essential metal for human health, acting as a cofactor that participates in more than 300 enzymatic reactions. It also plays a crucial role in the transmission of neural signals and neuromuscular coordination and can prevent neuronal excitation, contributing to overall neurodevelopmental protective effects.^10^ Currently, several studies on the association between dietary or supplemental Mg intake and neurodevelopment have shown that moderate Mg intake can promote neurodevelopment. A cross-sectional study from America, which assessed dietary intake through three 24-hour dietary recall interviews, demonstrated that lower dietary intake of Mg was associated with more callous-unemotional traits and antisocial behavior in children.^19^ In a randomized double-blind placebo-controlled trial, Mg and vitamin D co-supplementation was found to improve the behavioral function and mental health of children with ADHD.^33^ Furthermore, a meta-analysis indicated that serum and hair Mg levels were lower in children with ADHD than in healthy children.^34^ Another meta-analysis showed that serum Mg levels were negatively associated with the risk of ADHD.^18^ Similarly, our study found that increased Mg levels were associated with a reduced risk of neurodevelopmental delay, especially in the gross motor and personal-social domains. The protective effect of Mg remained significant in the essential metal mixture, further supporting the critical role of Mg in neurodevelopment.

Cu is an essential trace element that makes up human cells and is involved in many physiological processes, such as skin pigmentation, myelination, and neurotransmitter synthesis. Cu homeostasis is also critical in neurodevelopment.^12^ At present, there is inconclusive epidemiological evidence regarding the effects of Cu on neurodevelopment in children. A Polish cohort study of 539 mother-child pairs found no statistically significant association between Cu and neurodevelopment in children aged 1-2 years.^35^ However, a prospective cohort study in Spain revealed a negative association between prenatal Cu levels and neurodevelopment at 12 months of age in 651 children, particularly in boys.^20^ Other studies have suggested a positive effect of Cu on neurodevelopment. For example, a Chinese study provided evidence of an association between Cu and the neurodevelopment of 843 children aged 24 months, and observed a sex-specific pattern in which blood Cu levels were positively associated with the intelligence development index, and inversely U-shaped in association with the psychomotor development index in boys.^36^ The current study found a negative association between whole blood Cu levels and the risk of neurodevelopmental delay among preschool children, and the effect was more significant in the gross motor domain. Meanwhile, Cu still exhibited a protective effect in the essential metal mixture. The relationship between Cu and neurodevelopment requires further validation in a large prospective cohort study.

Zn mainly plays a major role in various physiological processes, such as cell growth, development, and differentiation. Furthermore, Zn is essential for promoting body growth and development, forming metalloenzymes, supporting immune system development and neurodevelopment. However, studies on dietary or supplemental Zn intake and neurodevelopmental status in children are still controversial. For instance, one Chinese case-control study found that a zinc-rich nutritional pattern was negatively associated with ADHD in children.^37^ Some randomized controlled trials have verified the efficacy of Zn supplementation, but the evidence for efficacy is inconsistent,^38–40^ and the dosage and form of administration varies widely from trial to trial, making the optimal administration strategy still unclear. Regarding the association between internal exposure to Zn and neurodevelopment, a cohort study in Nepal suggested no significant association between cord blood levels of Zn and neurodevelopment in children aged 36 months.^41^ Similarly, a meta-analysis including 11 observational studies found no statistically significant difference in blood and hair Zn levels between healthy and ADHD children.^17^ Another meta-analysis had similar findings.^42^ However, a case-control study in China found that whole blood Zn levels in preschool children with ASD were significantly reduced by 6% compared to the control group and that whole blood Zn levels were negatively associated with ASD.^21^ Additionally, a case-control study from Malaysia found that urinary Zn levels in children with ASD were significantly lower than those in healthy controls. Higher urinary Zn levels were associated with a reduced risk of ASD.^22^ Furthermore, our study found a negative association between elevated Zn levels and the risk of neurodevelopmental delay, supporting the protective effect of Zn in neurodevelopment. In addition, this protective effect was also sex-specific in our study, showing a statistically significant negative association in girls, which may be related to sexual differences in hormone secretion.^43^

The health effect of the essential metal mixture depends on the type of metal, the dose or concentration, the duration of exposure, and the joint effect of multiple metals, which can either enhance or diminish the overall health effects. Most studies have focused on the effects of interactions between heavy metals on neurodevelopment, while research on multiple exposures to essential metals and neurodevelopment among preschool children is still at an early stage. For instance, a cross-sectional study assessed the relationship between co-exposure to arsenic (As), mercury (Hg), lead (Pb), cadmium (Cd), selenium (Se) and intelligence quotient (IQ) among adolescents, and found a negative association between metal mixture and IQ, with As and Cd contributing the most to the mixture.^44^ A Bangladesh prospective birth cohort study which measured the concentrations of 52 trace elements in umbilical cord serum of 569 mother-infant pairs, evaluated the association of metal mixture with infant neurodevelopment and found that co-exposure to lithium (Li), aluminum (Al), and Fe was associated with lower cognitive composite scores, and that co-exposure to Zn, silver (Ag), and antimony (Sb) can affect motor composite scores.^45^ Our study initially analyzed the relationship between essential metal mixture (Mg, Fe, Cu, and Zn) and the neurodevelopment of preschool children, finding that the level of essential metal mixture was negatively associated with the risk of neurodevelopmental delay. Unfortunately, the current evidence regarding the mechanism of essential metal mixtures is insufficient. Therefore, further exploration is required to investigate the epidemiological and mechanistic association between essential metal mixture and neurodevelopment.

This study has several limitations. First, this study did not investigate other dietary and lifestyle factors that may influence the neurodevelopment of preschool children. However, the sensitivity analysis of the E-values showed that the results remained stable. Second, only the four most common essential metals in clinical practice were routinely measured, and the levels were assessed at a single time point, which is not representative of long-term exposure. Third, since ASQ-3 is a developmental screener, it may overestimate the prevalence of possible neurodevelopmental delay, thereby leading to an underestimation of the association between essential metals and neurodevelopmental delay. Finally, it should be noted that the preschool children were recruited from a single site located in the eastern part of China, which may lack the representativeness of our findings to children residing in other regions or countries. Future work should focus on large-scale multi-center studies for further validation.

## Conclusions

Our study found that the essential metals Mg, Cu, and Zn might play a protective role in reducing the risk of neurodevelopmental delay among preschool children, particularly in the gross motor and personal-social domains. The above conclusions need further validation in large-sample prospective cohort studies.

## Supplementary information

Supplementary Information

## Data Availability

The datasets used and/or analyzed during the current study are available from the corresponding author on reasonable request.
