# Supplementary material for: Association of whole blood essential metals with neurodevelopment among preschool children
Source: Pediatr Res. 2024 Nov 16;98(1):107–13. doi: 10.1038/s41390-024-03729-9 (PMC12411239; doi:10.1038/s41390-024-03729-9)
Supplement: Supplementary file 1 — Supplementary Information [file 41390_2024_3729_MOESM1_ESM.pdf]

Supplementary materials

Association of whole blood essential metals with neurodevelopment among preschool children

Ying Shen [1*], Wanting Zhang [2,3*], Huyi Jin [2], Fanjia Guo [2,4], Mingjuan Jin [2#], Guangdi Chen [2#]

1 Department of Child Health Care, Children's Hospital, National Clinical Research Center for Child Health, Zhejiang University School of Medicine, Hangzhou, 310005, China

2 Department of Public Health, Zhejiang University School of Medicine, Hangzhou, 310058, China

3 Jiaxing Center for Disease Control and Prevention, Jia Xing, 314050, China

4 Department of Environmental Health, Zhejiang Provincial Center for Disease Control and Prevention, Hangzhou, 310051, China

Ying Shen and Wanting Zhang are contributed equally.

Table S1 Reference intervals and distribution of essential metals

**Metal (Reference interval, μmol/L)**	**Overall (*n* = 4 487)**
Mg (1.12×10^3 - 2.06×10^3), *n* (%)	
< Lower limit of reference interval	2 (0.0)
Within reference interval	4,484 (99.9)
> Upper limit of reference interval	1 (0.0)
Fe (6.63×10^3 - 11.82×10^3), *n* (%)	
< Lower limit of reference interval	16 (0.4)
Within reference interval	4,471 (99.6)
> Upper limit of reference interval	0
Cu (11.80 - 39.30), *n* (%)	
< Lower limit of reference interval	0
Within reference interval	4,487 (100.0)
> Upper limit of reference interval	0
Zn [*], *n* (%)	
< Lower limit of reference interval	1,427 (31.8)
Within reference interval	3,059 (68.2)
> Upper limit of reference interval	1 (0.0)

[*] The age-specific reference intervals of Zn are: < 1 year, 58.00 - 100.00 μmol/L; < 2 years, 62.00 - 110.00 μmol/L; < 3 years, 66.00 - 120.00 μmol/L; < 5 years, 72.00 - 130.00 μmol/L; 5 years and above, 76.50 -150.00 μmol/L.

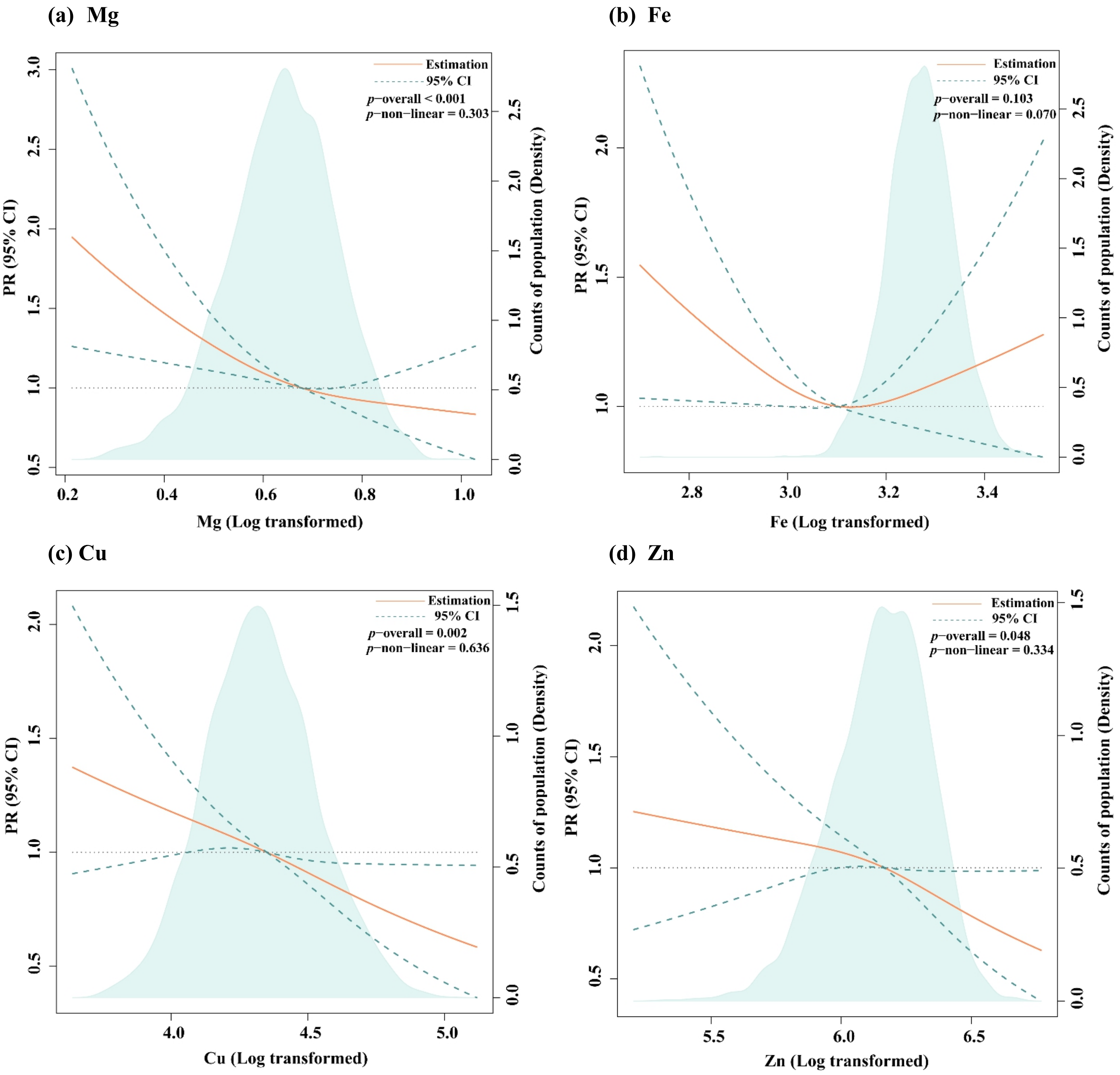

Fig. S1 The RCS model of the dose-response curves of essential metals and neurodevelopmental delay, adjusted for age, sex, and BMI. The solid lines represent the PRs based on RCS for the log-transformed levels of (a)Mg, (b)Fe, (c)Cu, and (d)Zn in the fully adjusted model with the reference value was set at the 10th percentile, and the dashed lines represent the 95% CIs. RCS, restricted cubic spline; PR, prevalence ratio; CI, confidence interval.

Table S2 Stratified analysis of the association between essential metal and the risk of neurodevelopmental delay, by age

**Age**	**< 1 year**	**< 2 years**	**< 3 years**	**< 4 years**	**4-5.5 years**
Delay / Typicality, *n*	98 / 457	181 / 1 064	150 / 682	81 / 694	152 / 928
PR (95% CI)*					
Mg					
Per $\log_2$ increment	0.21 (0.06-0.77)	0.84 (0.25-2.79)	0.09 (0.02-0.36)	1.53 (0.29-8.11)	0.29 (0.08-1.07)
T_1	1.00	1.00	1.00	1.00	1.00
T_2	0.84 (0.49-1.41)	0.68 (0.45-1.00)	0.67 (0.44-1.03)	0.94 (0.50-1.77)	0.98 (0.64-1.48)
T_3	0.52 (0.30-0.93)	0.93 (0.64-1.36)	0.44 (0.28-0.71)	1.43 (0.80-2.53)	0.85 (0.55-1.33)
$p_{\text{-trend}}$	0.032	0.657	< 0.001	0.245	0.489
Fe					
Per $\log_2$ increment	0.40 (0.13-1.26)	2.09 (0.59-7.41)	0.28 (0.06-1.40)	0.66 (0.10-4.37)	1.15 (0.26-5.11)
T_1	1.00	1.00	1.00	1.00	1.00
T_2	0.72 (0.42-1.23)	0.89 (0.60-1.33)	0.90 (0.58-1.40)	0.68 (0.37-1.24)	0.70 (0.45-1.08)
T_3	0.80 (0.47-1.36)	1.27 (0.87-1.86)	0.83 (0.53-1.31)	0.80 (0.46-1.40)	0.87 (0.58-1.32)
$p_{\text{-trend}}$	0.465	0.212	0.325	0.513	0.544
Cu					
Per $\log_2$ increment	0.42 (0.18-0.97)	0.95 (0.52-1.71)	0.39 (0.21-0.73)	0.70 (0.29-1.70)	0.57 (0.32-1.04)
T_1	1.00	1.00	1.00	1.00	1.00
T_2	0.62 (0.36-1.06)	0.85 (0.57-1.27)	0.85 (0.55-1.31)	0.72 (0.40-1.32)	0.73 (0.48-1.11)
T_3	0.60 (0.35-1.03)	0.99 (0.68-1.45)	0.55 (0.35-0.88)	0.85 (0.48-1.50)	0.61 (0.40-0.95)
$p_{\text{-trend}}$	0.088	0.967	0.009	0.475	0.030
Zn					
Per $\log_2$ increment	0.50 (0.20-1.22)	1.87 (0.86-4.07)	0.56 (0.22-1.45)	0.90 (0.27-3.02)	0.24 (0.09-0.63)
Low-level	1.17 (0.66-2.08)	1.06 (0.71-1.59)	1.27 (0.80-2.03)	0.91 (0.50-1.67)	1.32 (0.86-2.02)
≤ Median	1.00	1.00	1.00	1.00	1.00
> Median	1.07 (0.54-2.13)	1.47 (0.99-2.18)	1.02 (0.66-1.57)	0.92 (0.52-1.61)	0.64 (0.42-0.97)
$p_{\text{-trend}}$	0.909	0.059	0.938	0.662	0.035

* The prevalence ratios were estimated by modified Poisson regression, adjusted for sex and BMI.

PR, prevalence ratio; CI, confidence interval; T_1, 1st tertile; T_2, 2nd tertile; T_3, 3rd tertile.

Table S3 Stratified analysis of the association between essential metal and the risk of neurodevelopmental delay, by sex

**Sex**	**Boy**	**Girl**
Delay / Typicality, *n*	451 / 2 134	211 / 1 691
PR (95% CI)*		
Mg		
Per log_2 increment	0.45 (0.22-0.92)	0.21 (0.08-0.58)
T_1	1.00	1.00
T_2	0.85 (0.67-1.09)	0.72 (0.51-1.02)
T_3	0.78 (0.60-1.00)	0.73 (0.51-1.03)
$p_{\text{-trend}}$	0.050	0.067
Fe		
Per log_2 increment	1.04 (0.47-2.28)	0.36 (0.13-0.99)
T_1	1.00	1.00
T_2	0.92 (0.72-1.19)	0.70 (0.49-1.00)
T_3	0.97 (0.76-1.26)	0.72 (0.50-1.03)
$p_{\text{-trend}}$	0.843	0.090
Cu		
Per log_2 increment	0.63 (0.43-0.92)	0.47 (0.28-0.78)
T_1	1.00	1.00
T_2	0.90 (0.70-1.14)	0.54 (0.38-0.77)
T_3	0.76 (0.59-0.98)	0.64 (0.46-0.90)
$p_{\text{-trend}}$	0.031	0.017
Zn		
Per log_2 increment	0.72 (0.43-1.21)	0.49 (0.25-0.98)
Low-level	1.13 (0.88-1.45)	0.85 (0.61-1.19)
≤ Median	1.00	1.00
> Median	0.96 (0.72-1.28)	0.48 (0.31-0.75)
$p_{\text{-trend}}$	0.865	0.009

* The prevalence ratios were estimated by modified Poisson regression, adjusted for age and BMI.

PR, prevalence ratio; CI, confidence interval; T_1, 1st tertile; T_2, 2nd tertile; T_3, 3rd tertile.

Table S4 Stratified analysis of the association between essential metal and the risk of neurodevelopmental delay, by BMI

**BMI**	**Underweight**	**Normal**	**Overweight**	**Obesity**
Delay / Typicality, *n*	39 / 177	515 / 3 142	75 / 318	33 / 188
PR (95% CI)*				
Mg				
Per $\log_2$ increment	0.35 (0.02-5.50)	0.37 (0.19-0.71)	0.14 (0.02-0.74)	0.83 (0.04-16.41)
T_1	1.00	1.00	1.00	1.00
T_2	0.79 (0.34-1.86)	0.80 (0.64-1.00)	0.68 (0.37-1.24)	0.63 (0.25-1.61)
T_3	0.79 (0.33-1.91)	0.78 (0.62-0.98)	0.60 (0.30-1.19)	0.71 (0.29-1.75)
$p_{\text{-trend}}$	0.590	0.030	0.134	0.408
Fe				
Per $\log_2$ increment	0.58 (0.01-23.03)	0.87 (0.43-1.75)	0.20 (0.03-1.55)	1.05 (0.09-12.04)
T_1	1.00	1.00	1.00	1.00
T_2	1.24 (0.51-3.00)	0.86 (0.68-1.08)	0.52 (0.28-0.95)	1.34 (0.51-3.52)
T_3	0.90 (0.35-2.35)	0.95 (0.75-1.20)	0.47 (0.26-0.88)	1.36 (0.54-3.42)
$p_{\text{-trend}}$	0.826	0.658	0.022	0.497
Cu				
Per $\log_2$ increment	0.90 (0.25-3.31)	0.54 (0.39-0.75)	0.56 (0.20-1.56)	0.71 (0.13-3.75)
T_1	1.00	1.00	1.00	1.00
T_2	0.91 (0.39-2.14)	0.76 (0.60-0.95)	0.73 (0.38-1.41)	0.49 (0.19-1.27)
T_3	0.98 (0.40-2.39)	0.69 (0.55-0.87)	0.83 (0.44-1.55)	0.68 (0.28-1.65)
$p_{\text{-trend}}$	0.987	0.002	0.550	0.412
Zn				
Per $\log_2$ increment	0.33 (0.06-1.81)	0.60 (0.38-0.96)	0.73 (0.17-3.16)	1.65 (0.37-7.28)
Low-level	1.39 (0.57-3.41)	1.05 (0.84-1.31)	0.98 (0.52-1.85)	1.20 (0.46-3.12)
≤ Median	1.00	1.00	1.00	1.00
> Median	0.47 (0.17-1.26)	0.80 (0.61-1.05)	1.42 (0.71-2.84)	1.43 (0.37-5.50)
$p_{\text{-trend}}$	0.171	0.291	0.500	0.644

* The prevalence ratios were estimated by modified Poisson regression, adjusted for age and sex.

BMI, body mass index; PR, prevalence ratio; CI, confidence interval; T_1, 1st tertile; T_2, 2nd tertile; T_3, 3rd tertile.
